# Identification of the effects of COVID-19 on patients with pulmonary fibrosis and lung cancer: a bioinformatics analysis and literature review

**DOI:** 10.1038/s41598-022-20040-x

**Published:** 2022-09-26

**Authors:** Yang Li, Lipeng Niu

**Affiliations:** 1 Shihua Residential District Community Health Service Center, Jinshan District, Shanghai, 201500 China; 2 Affiliated Hospital of NCO School, Army Medical University, Shijiazhuang, 050000 China

**Keywords:** Computational biology and bioinformatics, Cancer

## Abstract

Coronavirus disease 2019 (COVID-19) poses a serious threat to human health and life. The effective prevention and treatment of COVID-19 complications have become crucial to saving patients’ lives. During the phase of mass spread of the epidemic, a large number of patients with pulmonary fibrosis and lung cancers were inevitably infected with the SARS-CoV-2 virus. Lung cancers have the highest tumor morbidity and mortality rates worldwide, and pulmonary fibrosis itself is one of the complications of COVID-19. Idiopathic lung fibrosis (IPF) and various lung cancers (primary and metastatic) become risk factors for complications of COVID-19 and significantly increase mortality in patients. Therefore, we applied bioinformatics and systems biology approaches to identify molecular biomarkers and common pathways in COVID-19, IPF, colorectal cancer (CRC) lung metastasis, SCLC and NSCLC. We identified 79 DEGs between COVID-19, IPF, CRC lung metastasis, SCLC and NSCLC. Meanwhile, based on the transcriptome features of DSigDB and common DEGs, we identified 10 drug candidates. In this study, 79 DEGs are the common core genes of the 5 diseases. The 10 drugs were found to have positive effects in treating COVID-19 and lung cancer, potentially reducing the risk of pulmonary fibrosis.

## Introduction

SARS-CoV-2 is a novel coronavirus. It has directly contributed to the worldwide COVID-19 pandemic^1^. SARS-CoV-2 infection can have serious pulmonary fibrosis consequences^2^. IPF is a chronic progressive lung disease, nintedanib and pirfenidone are FDA-approved for the treatment of IPF. After a COVID-19 infection, survivors are likely to develop bilateral interstitial pneumonia, which often leads to acute respiratory distress syndrome and pulmonary fibrosis. A number of risk factors are shared by both IPF and lung cancer, and patients with both conditions have a worse prognosis than patients with either. In relation to the severity of IPF, the lung cancer stage matters more than the interval between diagnosis and lung cancer^3^. Patients with cancer appear to be particularly vulnerable to COVID-19, as tumors can severely affect the immunological response to viral infection^4^. Delays in screening, diagnosis and treatment due to the COVID-19 pandemic could lead to excess cancer deaths and delay or even reverse projected mortality declines for some cancers^5^; i.e., cancer patients with COVID-19 have a significantly higher mortality rate than infected patients without cancer^6^. As a result, patients with underlying diseases such as pulmonary fibrosis and lung cancer are more likely to suffer serious complications or even death after SARS-CoV-2 infection.

Patients with lung cancer, whose underlying lung function and endurance are poor, are more likely to experience more severe hypoxia and to progress more rapidly with COVID-19 infection, so treatment is urgently needed for lung cancer patients infected with COVID-19^7^. Of all lung cancers, small cell lung cancer (SCLC), although less common, is a rapidly fatal disease for which there is little effective clinical treatment^8^. Most patients will die within one year. Non-small cell lung cancer (NSCLC) is the most common type of lung cancer and although the death rate is lower than that of SCLC, it kills more people than SCLC because of the disproportionate number of cases^9^. In addition to primary lung cancer, secondary lung cancer caused by metastases from other types of cancer also constitutes an important risk factor after SARS-CoV-2 infection.

The lungs are the second most common site of metastasis from CRC after the liver. 11% of CRC patients present with isolated lung metastases and surgical removal of lung metastases is effective in some cases, but the prognosis is poor^10^. Surfactant protein D (SP-D) downregulates EGF signaling and inhibits lung cancer cell growth^11^. As an in vitro and in vivo test, SP-D was shown to suppress pulmonary metastases from CRC^12^.

In our study, five datasets were used to identify the biological relationships between COVID-19 and IPF, lung metastases from CRC, SCLC and NSCLC. First, differentially expressed genes were identified from the dataset, and then common differential genes were found for the five diseases. Using these common differential genes, further analyses, including enrichment analysis and pathway analysis, are performed to understand the biological processes involved in genome-based expression studies. PPI networks were made using common differential genes to collect the full range of hub genes. Common DEGs were also tracked according to GSE186460, GSE17978, GSE41258, GSE40275 and GSE33532 for transcriptional regulators. Finding the top 10 genes from hub genes is a key step in predicting potential drugs. Finally, an outlook on potential drugs for the hub gene is provided.

## Materials and methods

### Datasets used in this study

In order to obtain common genes between COVID-19, IPF, CRC lung metastasis, SCLC and NSCLC, we searched the GEO database from the NCBI (https://www.ncbi.nlm.nih.gov/geo/)^13^ to find datasets with both onset lung tissue and normal lung tissue controls and to download the full data. The GEO accession ID for the COVID-19 dataset is GSE186460^14^, which consists of 4 COVID-19 samples and 11 normal lung tissue samples sequenced by a high-throughput sequencing system called Illumina HiSeq 2500 (Homo sapiens) provided by Dobosh B et al. The GEO accession ID for the IPF dataset is GSE17978^15^, which includes 38 IPF samples and 20 normal lung tissue samples sequenced by the high-throughput sequencing system called Duke Human Operon 36 k v4.0 spotted microarray provided by Emblom-Callahan MC et al.; the GEO accession ID for the CRC lung metastasis dataset is GSE41258^16^, which includes 20 lung metastasis samples from CRC and 7 normal lung tissue samples sequenced by the high-throughput sequencing system called [HG-U133A] Affymetrix Human Genome U133A Array provided by Sheffer M et al. The GEO accession ID for the SCLC datasets GSE40275^17^, comprising 15 SCLC samples and 43 normal lung tissue samples, was sequenced by the high-throughput sequencing system called Human Exon 1.0 ST Array [CDF: Brainarray Version 9.0.1, HsEx10stv2_Hs_REFSEQ] provided by Kastner et al. The GEO accession ID of the NSCLC dataset is GSE33532^18^, including 80 NSCLC samples and 20 normal lung tissue samples, provided by Meister M et al. called [HG-U133_Plus_2] Affymetrix Human Genome U133 Plus 2.0 Array. See Table 1.

**Table 1:** A description of the datasets in this analysis together with their geo-features and quantitative measurements.

Disease name	GEO accession	GEO platform	Total DEGs count	Up regulated DEGs count	Down regulated DEGs count
COVID-19	GSE186460	GPL16791	7640	7211	429
IPF	GSE17978	GPL8903	2272	1420	852
NSCLC	GSE33532	GPL570	4407	2103	2304
SCLC	GSE40275	GPL15974	7164	4088	3076
CRC lung metastases	GSE41258	GPL96	2875	1387	1488

### Identification of differentially expressed genes in COVID-19, IPF, CRC lung metastasis, SCLC and NSCLC

A gene is described as differentially expressed when there are statistically significant differences between experimental conditions at the transcription level^19^. The key role of this analysis was to obtain DEGs for datasets GSE186460, GSE17978, GSE40275, GSE41258 and GSE33532. Using the R LIMMA package together with Benjamini-Hochberg procedure we have tackled the problem of multiple comparisons due to the DESEq2 analysis. The final list of DEGs were determined by applying the following thresholds: adj. *P*-value < 0.05 and |logFC| ≥ 1.0. Jvenn, an online VENN analysis tool, was used to gather mutual DEGs for GSE186460, GSE17978, GSE40275, GSE41258 and GSE33532^20^.

### Gene ontology (GO) and pathway enrichment analyses

Analyzing gene set enrichment is an important analytical exercise for classifying common biological insights, such as biological processes and chromosomal locations associated with different interlinked diseases^21^. Through EnrichR (https://maayanlab.cloud/Enrichr/)—a comprehensive gene set enrichment web tool, GO enrichment and functional enrichment (biological processes, cellular composition, and molecular functions) studies were conducted^22^ to characterize biological mechanisms and signaling pathways for shared DEGs. At the time, we used four databases, including KEGG (Kyoto Encyclopedia of Genes and Genomes)^23^, WikiPathways, Reactome, and BioCarta, as sources of pathway classification to identify shared pathways between COVID-19, IPF, CRC lung metastasis, SCLC, and NSCLC. Typically, the KEGG pathway controls metabolic processes and makes genome analysis quite useful. The P-value < 0.05 was considered a standard indicator for quantifying the highest pathways listed.

### PPI construction

Proteins conclude their journey into a cell with a similar protein affiliation formed by a Protein–Protein Interaction network, which indicates the protein mechanisms. In cell and systems biology, assessment and analysis of PPI networks and their functionalities are fundamental and key objectives for interpreting and gaining insight into cellular machinery operations. Using the STRING (https://string-db.org/) (Version 11.5)^24^ repository, we construct a protein PPI network from shared DEGs to describe functional and physical interactions between COVID-19, IPF, CRC lung metastasis, SCLC, and NSCLC. STRING envisions expanding PPI awareness through active interactive channels, including text mining, experimental databases, coexpression, culture, gene fusion, and coexistence under different taxonomic confidence levels (low, medium, and high). Moderate confidence was set at 0.5 to generate a common DEG PPI network. We then consumed our PPI network into Cytoscape (v.3.8.2) for visualization and further experimental PPI network studies. Cytoscape (v.3.8.2)-an open-source web visualization platform-serves as a flexible tool for combining multiple datasets to improve the performance of different interactions such as PPI, genetic interactions, and protein-DNA interactions^25^.

### Hub gene extraction and submodule analysis

A PPI network consists of nodes, edges, and their connections, where hub genes are the most entangled nodes. Cytohubba (http://apps.cytoscape.org/apps/Cytohubba)-a novel Cytoscape-plugin for ranking and extracting central or potential or target elements of biological networks based on various network characteristics. Cytohubba has 11 methods of investigating networks from different angles, with Maximal Clique Centrality (MCC) being the best of them^26^. Using the MCC method of Cytohubba, we identified the top 10 hub genes in the PPI network. Based on the proximity ranking characteristics of Cytohubba, we also categorized the shortest available pathways across hub genes.

### Evaluation of the applied medicine

In this study, one of the most important aspects is the prediction of protein-drug interactions (PDI) or the identification of drug molecules. Drug molecules were identified by Enrichr using the Drug Signatures database (DSigDB) based on COVID-19, IPF, CRC lung metastasis, SCLC, and NSCLC. The Enrichr web portal is one of the most popular online resources to explore the enrichment of gene sets across a genome-wide scale. DSigDB is the global archive for the reidentification of targeted drugs associated with DEGs^27^. The database has 22,527 gene sets, and access to the DSigDB database is via Enrichr in disease/ drug function.

### Ethics approval

Our study did not require ethical board approval because it did not contain human or animal trials. GEO belongs to public databases. The patients involved in the database have obtained ethical approval. Users can download relevant data for free for research and publish relevant articles. Our study is based on open source data, so there are no ethical issues and other conflicts of interest.

## Results

### Identification of common DEGs between COVID-19, IPF, CRC lung metastasis, SCLC and NSCLC

To explore the interrelationships and significance of COVID-19, IPF, CRC lung metastasis, SCLC, and NSCLC, based on the human RNA-seq and microarray datasets from the NCBI, we identified dysregulated genes that stimulate COVID-19, IPF, CRC lung metastasis, SCLC, and NSCLC. The RNA-seq and microarray dataset experiments were performed in an R-language environment characterized by DESeq2 and limit packs with Benjamin-Hochberg's false discovery rate.

COVID-19 has 7640 differential genes, of which 7211 are up-regulated and 429 are down-regulated; similarly, IPF has 2272 differential genes, of which 1420 are up-regulated and 852 are down-regulated; lung metastases from CRC have 2875 differential genes, of which 1387 are up-regulated and 1488 are down-regulated. SCLC has a total of 7164 differential genes, of which a total of 4088 are up-regulated and 3076 are down-regulated; NSCLC has a total of 4407 differential genes, of which a total of 2103 are up-regulated and 2304 are down-regulated.

**Figure 1:**
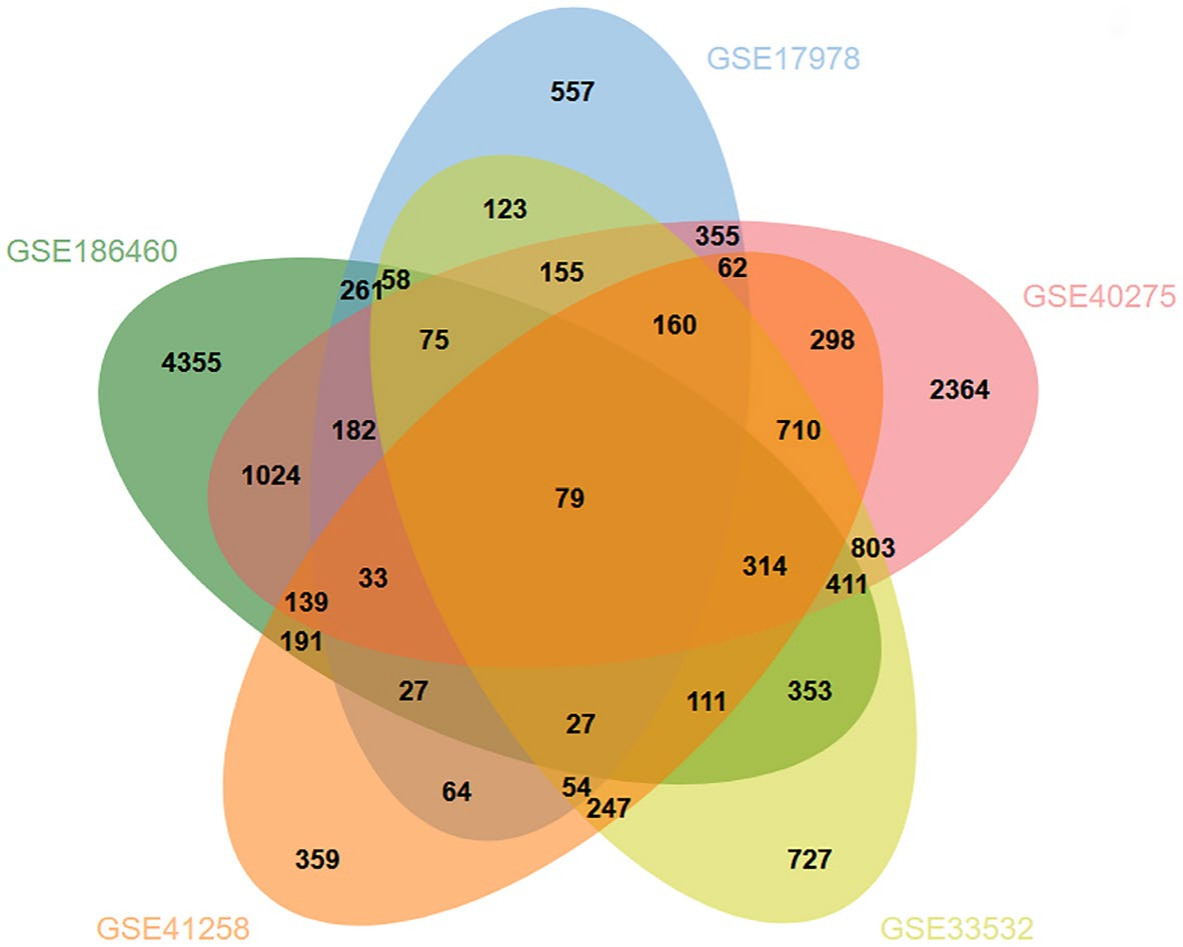
Among the datasets included in this study, IPF (GSE17978), COVID-19 (GSE186460), SCLC (GSE40275), NSCLC (GSE33532), and CRC lung metastasis (GSE41258) are analyzed using microarrays and RNA-seq. Based on this integrated analysis, we discovered 79 DEGs that are common among IPF, COVID-19, SCLC, NSCLC, and CRC lung metastases.

All significant DEGs were extracted at *P*-value < 0.05 and |logFC|≥ 1. After cross-referencing Jvenn (a reliable Venn analytics web portal), we identified 79 common DEGs from COVID-19, IPF, CRC lung metastasis, SCLC, and NSCLC datasets. This common set of genes was used to complete further experiments. The five diseases are linked because they share one or more genes. Figure 1 shows cumulative comparative evaluation and mutual DEGs retrieval of the five datasets.

### GO and pathway enrichment analyses

**Figure 2:**
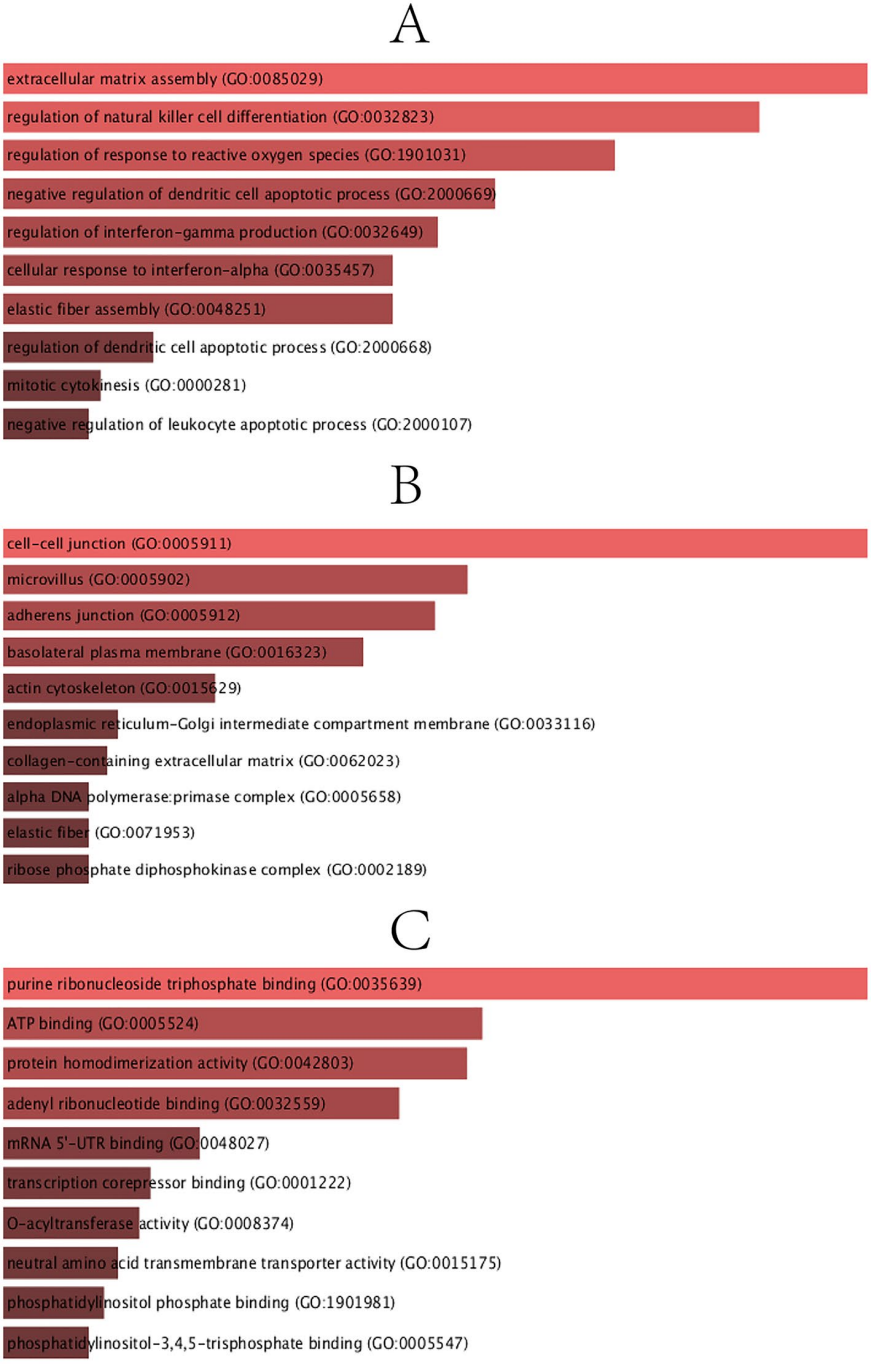
The bar graphs of ontological analysis of shared DEGs among IPF, COVID-19, SCLC, NSCLC, and CRC lung metastases performed by the Enrichr online tool: here, (**A**) Biological Processes, (**B**) Cellular Component, and (**C**) Molecular Function.

To determine the biological importance, pathway enrichment, and sharing of the DEGs highlighted in this study. GO and pathway enrichment analysis was carried out using Enrichr. Enrichr has a potentially improved method to compute enrichment, and the researchers demonstrated that this method might be better than the currently widely used Fisher exact test^22^. We have corrected the results for GO and Pathways using the improved method (Enrichr) to take the multiple comparison problem. GO, taking into account gene function and its composition provides a wide range of extensive computable knowledge resources for humans. Ontologies define theoretically defined bodies of information. An ontology and annotation serve to perform a detailed biological structural model, primarily for biological applications. The GO analysis was performed in terms of biological processes, cellular composition, and molecular function, and the GO database was selected as an annotation source. The top 10 terms for biological processes, molecular functions and cell composition categories are summarized in Table 2. Figure 2 also describes the linear features of the overall ontological analysis of each category in a bar graph.

**Table 2:** A descriptive analysis of the DEGs that are common to IPF, COVID-19, SCLC, NSCLC, and Colon cancer lung metastases.

Category	GO ID	Term	*P*-values	Genes
GO Biological Process	GO:0,085,029	Extracellular matrix assembly	1.13E-04	MFAP4;LTBP3;GAS6
	GO:0,032,823	Regulation of natural killer cell differentiation	1.53E-04	AXL;GAS6
	GO:1,901,031	Regulation of response to reactive oxygen species	2.29E-04	DHFR;STK26
	GO:2,000,669	Negative regulation of dendritic cell apoptotic process	3.19E-04	AXL;GAS6
	GO:0,032,649	Regulation of interferon-gamma production	3.74E-04	SLC7A5;AXL;GAS6;IL18R1
	GO:0,035,457	Cellular response to interferon-alpha	4.25E-04	AXL;GAS6
	GO:0,048,251	Elastic fiber assembly	4.25E-04	MFAP4;LTBP3
	GO:2,000,668	Regulation of dendritic cell apoptotic process	8.28E-04	AXL;GAS6
	GO:0,000,281	Mitotic cytokinesis	9.59E-04	KIF4A;MYH10;CEP55
	GO:2,000,107	Negative regulation of leukocyte apoptotic process	9.91E-04	AXL;GAS6
GO Cellular Component	GO:0,005,911	Cell–cell junction	9.79E-05	TMEM47;CEACAM1;CADM1;PTPRM;FLNA;PLPP3;RND1
	GO:0,005,902	Microvillus	0.001488612	SLC7A5;MYO1B;MSN
	GO:0,005,912	Adherens junction	0.001858139	CEACAM1;PTPRM;PLPP3;RND1
	GO:0,016,323	Basolateral plasma membrane	0.003025032	SLC7A5;CADM1;EPCAM;PLPP3
	GO:0,015,629	Actin cytoskeleton	0.00829552	MYO1B;AXL;FLNA;LPXN;RND1
	GO:0,033,116	Endoplasmic reticulum-Golgi intermediate compartment membrane	0.016068342	SERPINA1;PLPP3
	GO:0,062,023	Collagen-containing extracellular matrix	0.017279767	MFAP4;SERPINA1;ADAMTS1;HDGF;LTBP3
	GO:0,005,658	Alpha DNA polymerase:primase complex	0.019596385	PRIM1
	GO:0,071,953	Elastic fiber	0.019596385	MFAP4
	GO:0,002,189	Ribose phosphate diphosphokinase complex	0.019596385	PRPS2
GO Molecular Function	GO:0,035,639	Purine ribonucleoside triphosphate binding	8.24E-05	PRPS2;RASL12;RRM1;MYO1B;SNRK;STK26;SCG5;MYH10;RND1
	GO:0,005,524	ATP binding	8.13E-04	PRPS2;RRM1;MYO1B;SNRK;STK26;MYH10
	GO:0,042,803	Protein homodimerization activity	8.90E-04	GGCT;PRPS2;CEACAM1;PLN;CADM1;ZBTB16;STK26;RPE;FLNA
	GO:0,032,559	Adenyl ribonucleotide binding	0.001331367	PRPS2;RRM1;MYO1B;SNRK;STK26;MYH10
	GO:0,048,027	mRNA 5'-UTR binding	0.004357181	MYH10;CCT5
	GO:0,001,222	Transcription corepressor binding	0.005836836	ZBTB16;HDGF
	GO:0,008,374	O-acyltransferase activity	0.006237826	LPCAT1;PLA2G4A
	GO:0,015,175	Neutral amino acid transmembrane transporter activity	0.00707639	SLC7A5;SLC1A4
	GO:1,901,981	Phosphatidylinositol phosphate binding	0.00769414	MYO1B;ARAP3;PLA2G4A
	GO:0,005,547	Phosphatidylinositol-3,4,5-trisphosphate binding	0.008424198	MYO1B;ARAP3

**Figure 3:**
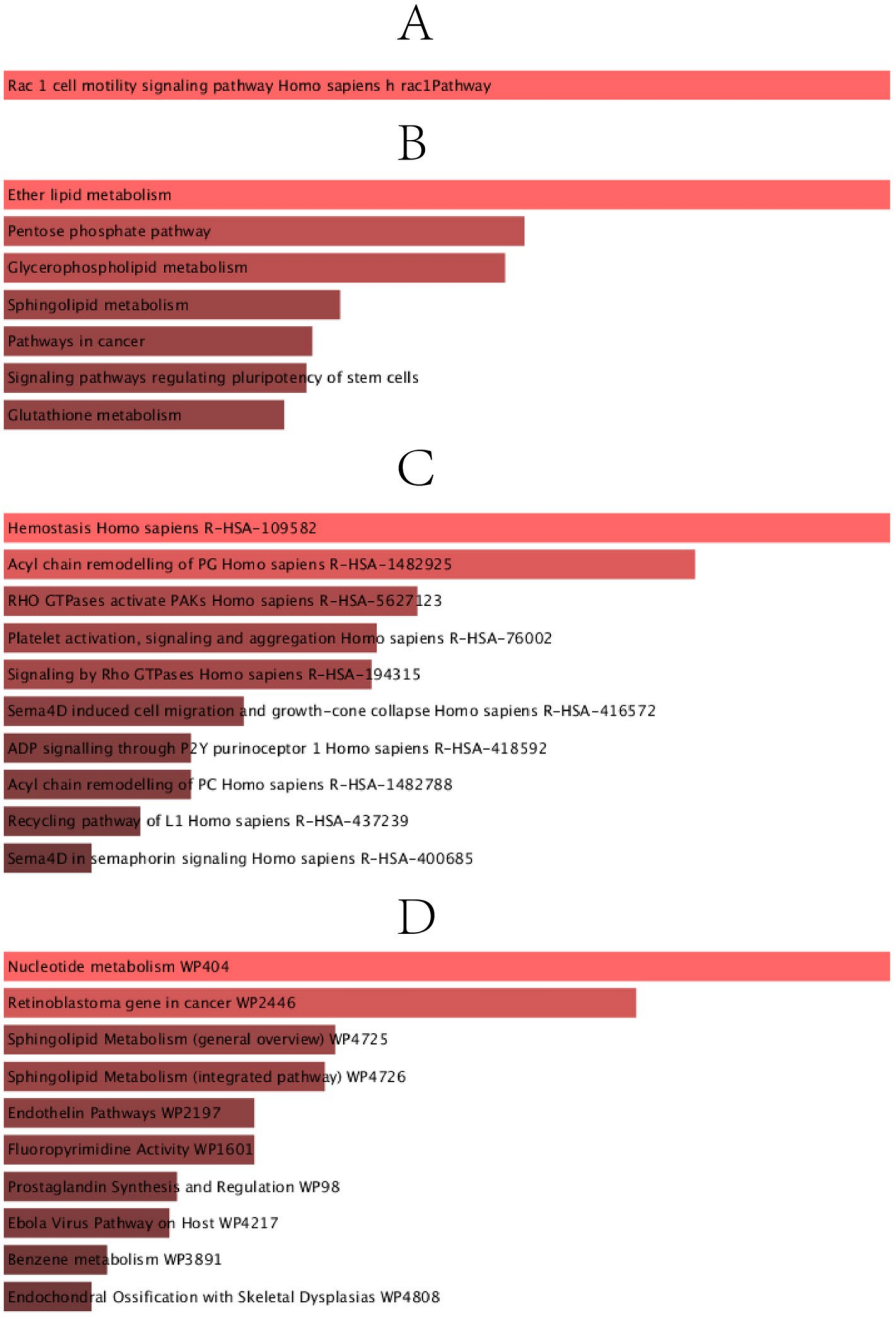
The bar graphs of pathway enrichment analysis of shared DEGs among IPF, COVID-19, SCLC, NSCLC, and CRC lung metastases performed by the Enrichr online tool: here, (**A**) BioCarta pathway, (**B**) KEGG pathway, (**C**) Reactome pathway, and (**D**) Wiki pathway.

Pathways analysis reveals how organisms respond to their inherent modifications. It is a model technique that demonstrates the interaction between diseases through fundamental molecular or biological processes. The most affected paths of the COVID-19, IPF, CRC lung metastasis, SCLC, and NSCLC joint DEGs were collected from four global databases, including KEGG, WikiPathways, Reactome and BioCarta. Table 3 shows the main paths from the selected datasets. In Fig. 3, the pathway enrichment analysis is also shown as bar graphs to illustrate this better.

**Table 3:** Analysis of pathway enrichment among IPF, COVID-19, SCLC, NSCLC, and CRC lung metastases.

Category	Pathways	*P*-values	Genes
BioCarta	Rac 1 cell motility signaling pathway Homo sapiens h rac1Pathway	0.008897062	CADM1;CHN1
KEGG	Ether lipid metabolism	9.59E-04	LPCAT1;PLA2G4A;PLPP3
	Pentose phosphate pathway	0.006237826	PRPS2;RPE
	Glycerophospholipid metabolism	0.006892716	LPCAT1;PLA2G4A;PLPP3
	Sphingolipid metabolism	0.016068342	CERS6;PLPP3
	Pathways in cancer	0.018517416	EDNRB;GNG4;ZBTB16;IL7R;CKS1B;RASGRP3
	Signaling pathways regulating pluripotency of stem cells	0.019064495	ID4;ID3;TBX3
	Glutathione metabolism	0.021369678	GGCT;RRM1
Reactome	Hemostasis Homo sapiens R-HSA-109582	0.001496763	SLC7A5;CEACAM1;SERPINA1;GNG4;KIF4A;PLA2G4A;FLNA;GAS6
	Acyl chain remodelling of PG Homo sapiens R-HSA-1482925	0.002016063	LPCAT1;PLA2G4A
	RHO GTPases activate PAKs Homo sapiens R-HSA-5627123	0.003081354	FLNA;MYH10
	Platelet activation, signaling and aggregation Homo sapiens R-HSA-76002	0.003278966	SERPINA1;GNG4;PLA2G4A;FLNA;GAS6
	Signaling by Rho GTPases Homo sapiens R-HSA-194315	0.003306344	CHN1;ARAP3;FLNA;MYH10;NDC80;NUP37
	Sema4D induced cell migration and growth-cone collapse Homo sapiens R-HSA-416572	0.004018856	MYH10;RND1
	ADP signalling through P2Y purinoceptor 1 Homo sapiens R-HSA-418592	0.004357181	GNG4;PLA2G4A
	Acyl chain remodelling of PC Homo sapiens R-HSA-1482788	0.004357181	LPCAT1;PLA2G4A
	Recycling pathway of L1 Homo sapiens R-HSA-437239	0.004708244	KIF4A;MSN
	Sema4D in semaphorin signaling Homo sapiens R-HSA-400685	0.005071943	MYH10;RND1
Wiki	Nucleotide metabolism WP404	5.49E-05	PRPS2;DHFR;RRM1
	Retinoblastoma gene in cancer WP2446	3.91E-04	DHFR;RRM1;PRIM1;KIF4A
	Sphingolipid Metabolism (general overview) WP4725	0.004018856	CERS6;PLPP3
	Sphingolipid Metabolism (integrated pathway) WP4726	0.004357181	CERS6;PLPP3
	Endothelin Pathways WP2197	0.007513763	CNN1;EDNRB
	Fluoropyrimidine Activity WP1601	0.007513763	DHFR;RRM1
	Prostaglandin Synthesis and Regulation WP98	0.013664889	EDNRB;PLA2G4A
	Ebola Virus Pathway on Host WP4217	0.014520104	AXL;FLNA;GAS6
	Benzene metabolism WP3891	0.023469932	EPHX1
	Endochondral Ossification with Skeletal Dysplasias WP4808	0.026520035	ADAMTS1;CTSV

### Classification of hub genes and submodule construction

**Figure 4:**
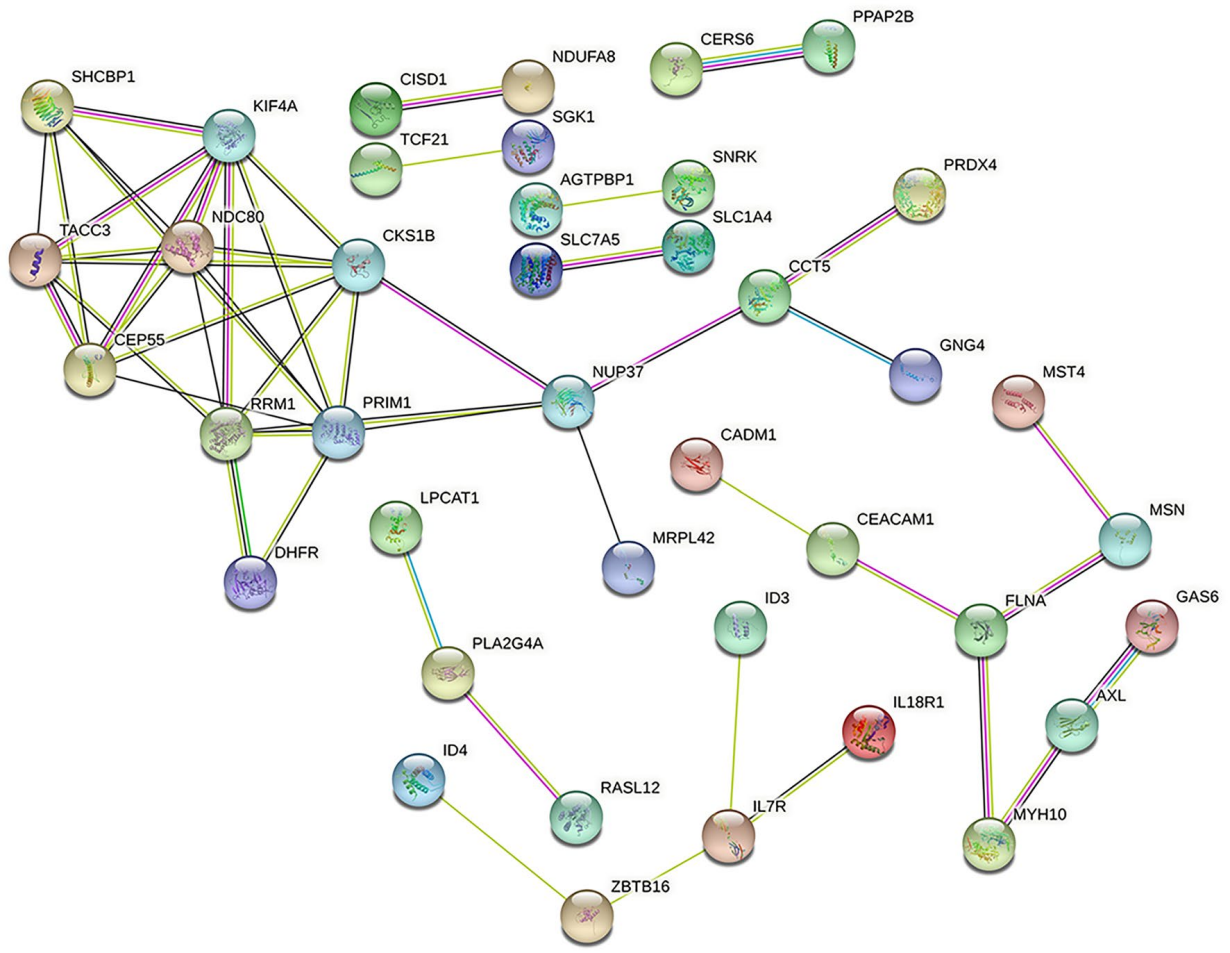
PPI network of common DEGs among IPF, COVID-19, SCLC, NSCLC, and CRC lung metastases.

**Figure 5:**
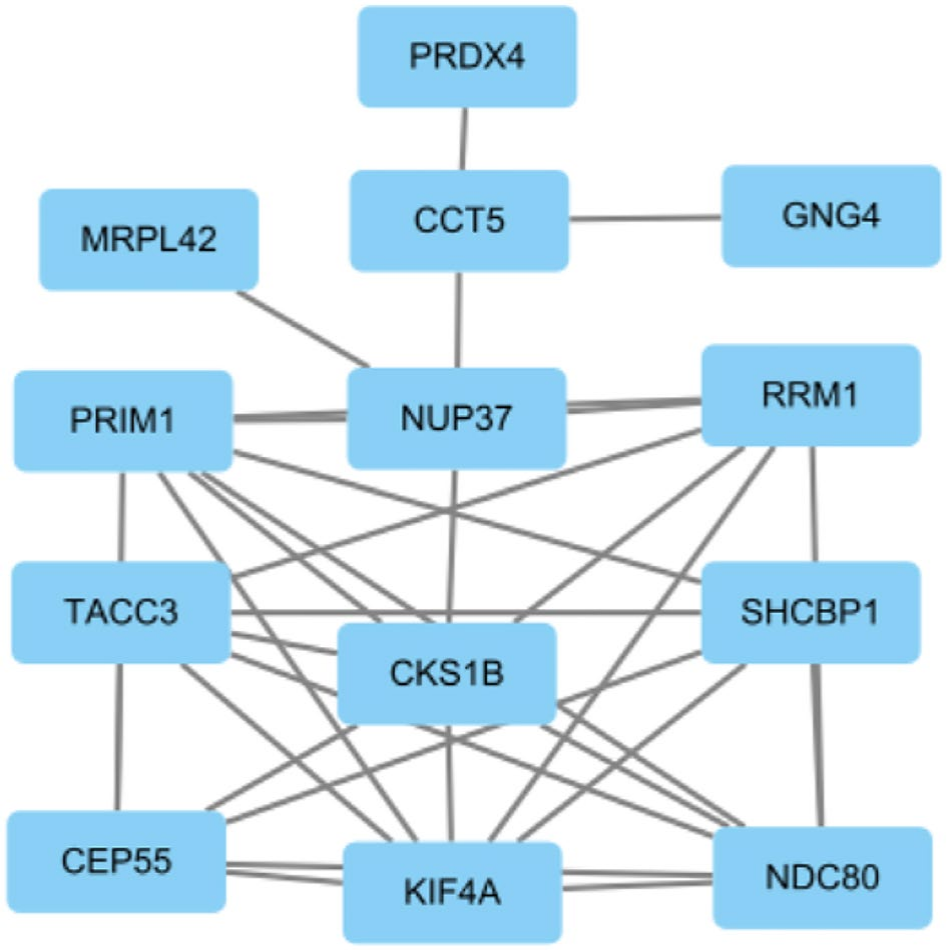
Determination of hub genes from the PPI network by using the Cytohubba plugin in Cytoscape. The latest MCC procedure of the Cytohubba plugin was pursued to obtain hub genes.

We used the online tool STRING to construct the protein network, the PPI network contains 79 nodes and 51 edges, see Fig. 4, and using the Cytohubba plugin in Cytoscape, we listed the 10 (10.87%) most influential genes as NDC80, KIF4A, CKS1B, CEP55, PRIM1, TACC3, RRM1, SHCBP1, NUP37, and CCT5. These hub genes could be potential biomarkers, which could also lead to new therapeutic strategies to treat the studied diseases. Since the central gene is latent, with the help of the Cytohubba plugin, we also constructed a submodule network (Fig. 5). By of Hub-gene interactions derived from the PPI network.

### Identification of candidate drugs

**Figure 6:**
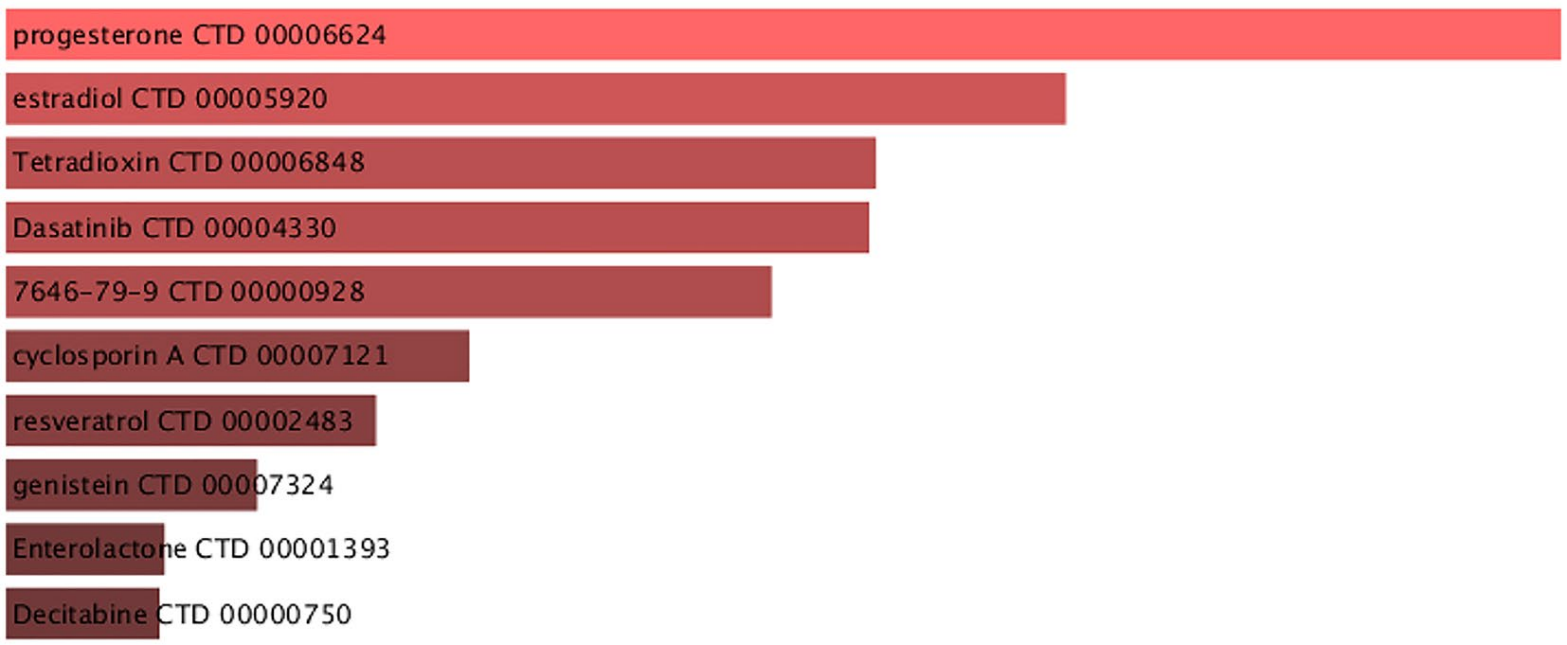
List of the suggested drugs among COVID-19, IPF, NSCLC, SCLC and CRC lung metastases.

Assessing protein-drug interactions is important for understanding the structural characteristics of receptor sensitivity. In conjoint DEGs for COVID-19, IPF, CRC lung metastasis, SCLC, and NSCLC, we identified 10 potential drug molecules based on transcriptome signatures of DSigDB; these potential drugs are recommended for corporate diseases; and they can be collective compounds for treating five diseases. Figure 6 shows the active agents of commonly used drugs in the DSigDB database. The criteria for candidate drugs screening are to select the top 10 in ascending order of Adjusted *P*-value (Table 4). Adjusted *P*-value < 0.05 was considered statistically significant.

**Table 4:** A list of candidate drugs that are common to IPF, COVID-19, SCLC, NSCLC, and CRC lung metastases.

Drugs	Adjusted *P*-value	Genes
progesterone CTD 00,006,624	1.22E-13	RASL12;PAK1IP1;SLC1A4
estradiol CTD 00,005,920	2.17E-10	COLEC12;RASL12;SERPINA1
Tetradioxin CTD 00,006,848	2.77E-09	COLEC12;RASL12; CCDC69
Dasatinib CTD 00,004,330	2.77E-09	RRM1;PRIM1;EPHX1
7646–79-9 CTD 00,000,928	1.11E-08	PRPS2;SERPINA1;WBP2
cyclosporin A CTD 00,007,121	1.36E-06	PRPS2;WBP2;PAK1IP1
resveratrol CTD 00,002,483	5.44E-06	PRPS2;RRM1;CERS6
genistein CTD 00,007,324	3.38E-05	SERPINA1;PRIM1;CCDC69
Enterolactone CTD 00,001,393	1.35E-04	RRM1;CADM1;PRIM1
Decitabine CTD 00,000,750	1.35E-04	RASL12;SERPINA1;CADM1

## Discussion

As the COVID-19 pandemic continues, the number of new COVID-19 cases continues to rise globally as the SARS-CoV-2 Omicron variant spreads^28^. Some experts predict that humans and COVID-19 may coexist in the long term^29^. Patients with cancer, including those with lung cancer, are more likely to contract SARS-CoV-2 regardless of vaccination status due to reduced immunity and to develop complications after infection. Patients with lung cancer are at high risk of hospitalization and death due to COVID-19^30^. Several non-coding RNAs that inhibit SARS-CoV-2 gene expression have been proposed to prevent multiple viral infections, pulmonary hypertension, and related diseases^31^.

In this paper, 10 hub genes related to five diseases of COVID-19, IPF, CRC lung metastasis, SCLC, and NSCLC were identified by means of bioinformatics, namely NDC80, KIF4A, CKS1B, CEP55, PRIM1, TACC3, RRM1, SHCBP1, NUP37 and CCT5. By analyzing these genes, the following related conclusions were obtained. TACC3 is involved in regulating normal cell growth and differentiation. Overexpression of TACC3 was previously associated with poor prognosis in lung cancer, and its expression levels were associated with clinical outcomes of lung cancer patients^32^. SHCBP1 plays an important role in the development of NSCLC^33^. The protein levels of NDC80, which is required for chromosome segregation, are kept low early in the cell cycle to allow the normal assembly of meiotic I kinetochores^34^. CEP55 protein levels are significantly reduced in extracellular vesicles from SDCBP-knockout lines^35^. RRM1 overexpression has been associated with gemcitabine resistance^36^. RRM1 can be used as a biomarker of gemcitabine resistance, and its increased expression has been shown to lead to gemcitabine resistance^37^. Furthermore, EGFR and KRAS are the most common mutated oncogenic drivers in lung adenocarcinoma^38^. While KRAS, HRAS and NRAS are all known oncogenes, the KRAS isoform is most commonly mutated in cancer, particularly in lung adenocarcinoma^39^. Studies on lung cancer have shown that deregulated expression or genomic alterations of KRAS oncogenes contribute to tumor progression and metastasis^40^. The absence of KEAP1 promoted metastasis in their lung cancer model expressing KRAS^41^.

The best way to understand how an organism responds is through its pathway analysis. A KEGG pathway containing 79 common DEGs has been identified as a similar pathway in five diseases. The top ten KEGG human pathways include ether lipid metabolism, pentose phosphate pathway, glycerophospholipid metabolism, sphingolipid metabolism, pathways in cancer, signaling pathways regulating pluripotency of stem cell, glutathione metabolism, TGF-beta signaling pathway, Fc gamma R-mediated phagocytosis and choline metabolism in cancer. Etheric lipid metabolism is strongly associated with lung cancer^42^.

We identified 10 drug candidates: progesterone, estradiol, tetradioxin, dasatinib, decitabine, 7646-79-9 (cobalt chloride), cyclosporine A, resveratrol alcohol, genistein, and enterolactone. These drugs may have potential activity in 5 diseases.

The anticancer agent dasatinib induces the apoptosis of cancer cells^43^. 5-Fu-induced apoptosis in CRC is significantly reduced by dasatinib by inhibiting Src activation^44^. TRAF6 depletion induces decitabine resistance in triple-negative breast cancer by blocking decitabine-induced DNA methyltransferase DEGs radiation^45^. Cyclosporin A is a powerful immunosuppressive agent that acts on T-lymphocytes and blocks effective T-cell receptor signaling^46,47^. By blocking MEK/ERK/c-Fos pathways, immunosuppressants like cyclosporine A, which is used to treat non-small lung cancer, reduce TRPM6 expression^48^. Resveratrol alcohol has shown promising activity in preventing and treating cancer^49^. Resveratrol inhibits cancer progression by inducing p53-dependent cell death^50^. Pancreatic cancer cells can be inhibited in their growth and apoptosis can be induced by Genistein by inhibiting oncogenic miR-223 expression^51^. There is strong evidence that higher serum enterolactone concentrations and increased lignan intake improve patients' prognoses after menopause^52^.

About 15 million people died in the first two years of the COVID-19 pandemic, according to new data from the World Health Organization (WHO). WHO estimates the global excess death toll in 2020 and 2021 at 14.9 million. Most of these deaths (84%) were concentrated in Southeast Asia, Europe and the Americas, and more than two-thirds (68%) occurred in just 10 countries^53^. Children infected with Omicron have a lower risk of serious illness than children infected with Delta^54^.

The current global COVID-19 pandemic is caused by the rapid international spread of SARS-CoV-2 infection^55–57^. In essence, COVID-19 can range from mild, self-limiting respiratory dis- ease to severe progressive pneumonia, multiorgan failure, and even death. SARS-CoV-2 infection, especially severe infection, is associated with an increased risk of longitudinal cognitive decline^58^. At the same time, SARS-CoV-2 has a high transmission rate, and there is a lack of adequate and effective treatments. Consequently, the increase in the number of respiratory distress cases has the potential to overwhelm global health care capacity^59^.

A recent study shows that tissue damage related to COVID-19 is mainly mediated by host innate immunity^60^. In critically ill COVID-19 patients, the host immune response is thought to play a key role in driving acute pneumonia with diffuse alveolar injury, inflammatory infiltration and microvascular thrombosis^61^. Expression of cell receptors (such as IFNGR1 and CXCR4) was reduced by a viral infection and is associated with suppression of associated signaling pathways and immune function^62^. A systematic evaluation in 2020 estimated that 28% of patients with severe COVID-19 had venous thromboembolism. Complications of COVID-19 thrombosis include arterial and venous events, and microvascular thrombosis may lead to diffuse alveolar damage, the main source of lung injury in COVID-19 patients^63,64^. Among COVID-19 patients, acute respiratory failure is the main reason for admission to the intensive care unit, and hypoxic respiratory failure is the most common life-threatening complication of COVID-19^65,66^.

In addition, in patients with COVID-19, ACE2 may play a causative role in cardiovascular complications such as thrombosis, heart damage and heart failure. ACE2 may be the link between SARS-CoV-2 and the cardiac manifestations identified in global data on the COVID-19 pandemic^67^. Recent studies have proven that COVID-19 is an independent risk factor for ischaemic stroke and acute myocardial infarction^68^. Additionally, older age is a significant independent predictor of mortality from SARS and Middle East respiratory syndrome (MERS). Similarly, increased age is associated with death among patients with COVID-19^69^. Many patients who die from COVID-19 are older and weaker with significantly compromised lung and/or immune function^70^.

Thus, to combat the COVID-19 pandemic, there is an urgent need to identify effective SARS-CoV-2 therapeutics that improve prognosis, particularly those with utility in the outpatient setting^71^. While new treatments are being developed, there is growing interest in repurposing existing drugs for COVID-19^72,73^. Aside from the recently licensed LY-CoV1404 (bebtelovimab), there are no licensed monoclonal antibody therapies that adequately target all Omicron variants^74^. Itaconic acid supplementation and CLYBL inhibition are possible therapeutic options for the treatment of COVID-19 that aim to modulate host defense to combat SARS-CoV-2 infection^75^. Hydroxychloroquine has been widely promoted as a potential treatment for COVID-19 due to its anti-inflammatory effects and antiviral activity in vitro studies^76^. Azithromycin is a widely used drug that may reduce viral load when combined with hydroxychloroquine in patients with non-severe COVID-19^77^. Trials of the JAK inhibitors baricitinib and ruxolitinib have shown promise in controlling excessive inflammation in COVID-19 patients^78^. Bamlanivimab monotherapy was reported to reduce the incidence of SARS-CoV-2 infection. Tocilizumab is an effective treatment for COVID-19 in patients with evidence of hypoxia and inflammation^79^.

Another strategy for combatting the global COVID-19 pandemic is to develop and produce COVID-19 vaccines^80^. Vaccination will be a key strategy for limiting the spread of SARS-CoV-2, reducing mortality, and controlling the COVID-19 pandemic^81,82^. The Pfizer-BioNTech vaccine is 95% effective against laboratory-confirmed COVID-19, and the Oxford–AstraZeneca vaccine was found to be 70% effective against COVID-19 in seronegative participants^83^.

Several guidelines issued at the start of the COVID-19 pandemic recommended delaying systemic anticancer treatment until COVID-19 symptoms had completely subsided^84^. However, failure to provide effective cancer treatment to many cancer patients during a pandemic increases cancer morbidity and mortality, which may be more serious than COVID-19 itself^85^. Patients with cancer are ostensibly more likely to develop and succumb to COVID-19 due to immunosuppression, increased comorbidity, and in the case of those with lung malignancies, potential pre-existing lung damage^86^.

Results from a recent study suggest that severe COVID-19 can lead to bilateral interstitial pneumonia, which usually results in acute respiratory distress syndrome and pulmonary fibrosis in survivors^87^. In essence, IPF is a chronic, progressive lung disease with a median survival time of 5.7 years^88^. Pulmonary fibrosis, similar to other types of fibrosis, is characterized by excessive accumulation of collagen and other matrix proteins, leading to distorting of pulmonary tissue structure and ultimately pulmonary failure^89^. Moreover, epithelial cell apoptosis and impaired autophagy are increasingly recognized as hallmarks of pulmonary fibrosis^90^. The causal role of endoplasmic reticulum stress in the pathogenesis of pulmonary fibrosis has been investigated^91^. The importance of CD4 + T cells producing interleukin-17A has been demonstrated in pulmonary fibrosis^92^.

Previously, IPF has been treated with a variety of chemical agents and drugs. For example, nintedanib and pirfenidone are FDA-approved for the treatment of IPF because they slow disease progression and improve lung function, exercise tolerance, and progression-free survival^93^. Compared to these two FDA-approved therapeutics for IPF, nebulized triiodothyronine has shown comparable or better effects on pulmonary fibrosis or survival rates^94^.

According to one study, those with IPF had a significantly higher chance of developing lung cancer than those of the same sex and age. Research supports that lung cancer is the leading cause of cancer-related deaths worldwide, with NSCLC accounting for 85% of cases^95^. The median overall survival (OS) and 5-year survival rates have historically been poor for patients with NSCLC^96^. The historical 5-year OS rate for locally advanced NSCLC patients treated with radical concurrent radiotherapy ranges from 25 to 30%^97^. As the leading cause of cancer-related deaths worldwide, adenocarcinoma of the lung accounts for approximately 50% of lung cancers, making it the most common subtype of NSCLC^98–100^. The highly aggressive NSCLC subtype squamous cell carcinoma of the lung accounts for one-third of all lung cancer cases^101,102^. The Keap1–Nrf2 pathway was associated with lung squamous cell carcinogenesis and chemoresistance^103,104^.

Besides, there is growing interest in the potential benefits of antimetastatic treatment for cancers such as lung adenocarcinoma, as a large number of patients are initially diagnosed with localized disease^105^. Maintenance therapy has become the standard of care for patients with advanced non-squamous NSCLC^106^. Of this total, approximately 50% of lung adenocarcinomas are molecularly subdivided, and their treatment depends on the presence of different molecular alterations, including EGFR mutations and ALK or ROS1 fusions, that confer sensitivity to selective kinase inhibitors^107^. For advanced EGFR-mutated lung adenocarcinoma, first-line therapy involves treatment with EGFR tyrosine kinase inhibitors^108^. There has been a study indicating that gene-based targeted therapy is the standard of care for patients with advanced NSCLC^109^. Atezolizumab monotherapy is effective in patients with PD-L1-selected advanced NSCLC^110^. In parallel, advances in immunotherapy have improved outcomes for a variety of cancers, including NSCLC^111^. For example, cancer immunotherapy with checkpoint inhibitors improves the survival rate of patients with NSCLC^112^. Based on the ability of immune checkpoint inhibitors to improve survival rates, these therapeutics have been approved for the treatment of a wide range of cancers, including NSCLC, melanoma, and uroepithelial cancer^113^. However, STK11 (LKB1) mutations are a major cause of primary resistance to immunotherapy in NSCLC^114^.

Surgical resection is currently the treatment option that provides the greatest long-term survival benefit for patients with early-stage NSCLC^115^. All studies suggest that pneumonectomy/metastasectomy is safe and can be performed in the absence of significant morbidity^116^. For patients with non-metastatic lung cancer, a proportion of patients can be cured after initial surgical resection, radiotherapy and/or a combination of therapeutic approaches^117^.

Oxaliplatin is a first-line treatment for breast cancer and NSCLC^118^. Triple-negative breast cancer is an aggressive form of invasive breast cancer defined by the lack of significant expression of the therapeutic target estrogen receptor, progesterone receptor and HER2^119^. circKIF4A is a prognostic biomarker and therapeutic target for triple-negative breast cancer^120^.

Several other studies have reported that loss-of-function mutations in the RB1 gene are common in several refractory cancers, such as SCLC and triple-negative breast cancer^121^. Among lung cancer cases, 15–30% are SCLC and one-third are diagnosed at the limited stage^122^. SCLC is an aggressive high-grade neuroendocrine malignancy and one of the deadliest solid tumors^123^. Among cancer patients, those with SCLC have one of the worst survival rates, with an overall 5-year survival rate of approximately 5%^124,125^. SCLC is characterized by a rapid doubling time and high growth fraction, and approximately two-thirds of patients present with metastases at the time of diagnosis^126^. SCLC is thought to acquire metastatic ability early during tumor progression. A previous study found that NFIB was highly expressed in more than 50% of human SCLC metastases, suggesting that upregulation of this transcription factor may be a driver of SCLC metastasis^127^. CREBBP plays a key role as a tumor suppressor in SCLC^128^.

SCLC is a rapidly fatal disease with few treatment options ^129^. The combination of a platinum drug and etoposide remains the mainstay treatment for SCLC^130^. However, cisplatin and etoposide improved progression-free survival (PFS) but failed to improve OS in patients with an extensive stage (ES)-SCLC^131^. The only FDA-approved drug for recurrent or progressive SCLC is topotecan, which has a response rate of 24% in patients with the platinum-sensitive disease and 2–6% in patients with platinum-refractory SCLC^132^. Although patients with SCLC usually respond initially to chemotherapy, the tumors almost always recur within 6–12 months, resulting in a 5-year survival rate of less than 7%^133^. Both DDR inhibition and immune checkpoint blockade are therapeutic strategies in preclinical and clinical development for patients with SCLC^134^. Patients with stage I to II SCLC have achieved long-term survival after radiotherapy with acceptable toxicity^135^. Suppression of DNA damage repair by poly [ADP-ribose] polymerase inhibitors have emerged as a potential therapeutic strategy for SCLC^136^. SCLC is sensitive to THZ1, a covalent CDK7 inhibitor with single-agent activity in T-cell acute lymphoblastic leukemia, MYCN- dependent neuroblastoma, and triple-negative breast cancer^137,138^. Pembrolizumab monotherapy is approved as a third-line or later therapy for metastatic SCLC^139^.

CRC is the second leading killer behind lung cancer, but must not be underestimated. CRC is one of the most common malignancies of the digestive system and the second most common cancer in the United States; in this country, CRC causes more than 50,000 deaths each year^140–142^. Dietary and lifestyle factors may significantly affect the risk of recurrence and death from CRC^143^. For example, obesity is associated with reduced survival among patients with metastatic CRC, particularly those receiving antiangiogenic therapy^144^.

COX-2 overexpression has been observed in a variety of malignancies, including lung and CRCs^145^. Aspirin may be more effective in preventing sporadic CRC in which COX-2 is over-expressed. In CRC, delayed initiation of chemotherapy is associated with a reduction in overall survival^146^. In patients with stage III CRC, adjuvant chemotherapy may improve OS^147^. Activation of p38-MAPK signaling due to KRAS mutation in CRC enables secondary colonization of the lungs by established liver metastases^148^.

The specific EGFR inhibitor cetuximab has been used to treat metastatic CRC, metastatic NSCLC and head and neck cancer^149^. Human metastatic lung cancer has high levels of HO1 and Bach1; thus, HO1 inhibitors represent an effective therapeutic strategy to prevent lung cancer metastasis^150^.

Since the World Health Organization declared a global pandemic on 11 March 2020, SARS- CoV-2 has caused more than 6.2 million deaths worldwide^151^. Compared to infection with the Delta variant, with Omicron carries a significantly lower risk of serious outcomes, with a greater reduction in the risk of more serious endpoints but the significant variation with age^152^. Since the beginning of the pandemic, more than 400 million cases of SARS-CoV-2 infection have been confirmed, including in people with chronic diseases such as cancer, diabetes and heart disease. These people are at high risk of serious illness and death associated with SARS-CoV-2 infection due to poor immune function as a result of pre-existing disease. The ultimate aim of this study was to improve the survival rate of patients with chronic diseases who are infected with SARS-CoV-2.

The COVID-19 pandemic poses an unprecedented challenge to global healthcare resources^153^. The pandemic has severely crowded out medical resources, and deaths from other diseases, such as CRC, have risen sharply. There are two main targets of CRC metastasis, the liver and lungs, but liver metastases are the most common. The liver has the considerable regenerative capacity and can regrow after partial surgical resection, whereas the lungs have no regenerative capacity, and lung function is irreversibly impaired after lung lobe removal due to CRC metastasis. The respiratory system is overwhelmed when patients with CRC lung metastasis become infected with SARS- CoV-2.

Recent studies have shown that a network where nodes are people and edges represent their social connections can effectively mimic the spreading of the virus^154^. Some graphics-based epidemiological models to simulate disease production and transmission pathways can significantly improve disease transmission control^155^.

There are many limitations to the treatment of cancer. One flaw of chemotherapy drugs is the inability to distinguish between malignant and normal cells; although chemotherapeutics kills cancer cells, they also non-selectively kill normal stem cells that must divide to maintain tissue homeostasis. In contrast, targeted drugs affect specific lesions, accumulating at the target site or releasing an active ingredient at the target site. However, mutation of the target renders the targeted drug ineffective, and drug resistance can occur with the long-term application. Immunotherapy aims to activate the host immune system, relying on its function to kill cancer cells and debulk tumors; this treatment approach is currently only effective in some cancers, and long-term treatment carries the risk of the cytokine storm.

Chemotherapy, targeted therapy and immunotherapy essentially kill cancer cells but have no effect on SARS-CoV-2. Only symptomatic treatment for COVID-19 is available, and eliminating the infection is dependent on the host immune system. Chemotherapy, targeted therapy and immunotherapy suppress immune function in cancer patients to varying degrees. If these patients are infected with SARS-CoV-2 while on treatment, their immune function will decrease drastically or even collapse, leading to a variety of serious complications and eventually systemic organ failure and death.

As mentioned above, although some specific drugs against SARS-COV-2 have been successfully developed, it is unclear whether they can have a positive effect in the treatment of lung cancer. People are more willing to discover and adopt existing drugs that are now well established to treat covid-19 and its complications. This article uses bioinformatics methods to screen out the 10 most meaningful drugs that act together on these five diseases. We found that these drugs have a positive effect in treating COVID-19 and lung cancer, potentially reducing the risk of pulmonary fibrosis caused by COVID-19. During the COVID-19 pandemic, in-depth research on these drugs may have certain reference significance for the prevention and treatment of complications in lung cancer patients infected with SARS-COV-2.

## Conclusion

In this study, we identified 79 DEGs between COVID-19, IPF, CRC lung metastasis, SCLC and NSCLC. In our opinion, these DEGs are the common core genes of the 5 diseases. Meanwhile, based on the transcriptome features of DSigDB and common DEGs, we identified 10 drug candidates. The treatment of COVID-19 and lung cancer with these drugs may show positive results, potentially reducing the risk of COVID-19-induced pulmonary fibrosis. This research provides new ideas that have not yet been experimentally validated, and additional scientific research is needed to validate our findings and hypotheses.

## Data Availability

The datasets used and/or analyzed during the current study are available from the corresponding author upon reasonable request. The datasets (GSE186460, GSE17978, GSE41258, GSE40275 and GSE33532) analyzed during the current study are available in the Gene Expression Omnibus (GEO) repository (http://www.ncbi.nlm.nih.gov/geo/).

## Abbreviations

COVID-19: Coronavirus disease 2019
SARS-CoV-2: Syndrome coronavirus 2
SCLC: Small cell lung cancer
NSCLC: Non-small cell lung cancer
IPF: Idiopathic lung fibrosis
CRC: Colorectal cancer
BP: Biological process
CC: Cellular component
DEGs: Differentially expressed gene
KEGG: Kyoto encyclopedia of genes and genomes
MF: Molecular function
PPI: Protein- Protein Interaction
EGFR: Epidermal growth factor receptor
SP-D: Surfactant protein D
